# A capillary electrophoresis coupled to mass spectrometry pipeline for long term comparable assessment of the urinary metabolome

**DOI:** 10.1038/srep34453

**Published:** 2016-10-03

**Authors:** Franck Boizard, Valérie Brunchault, Panagiotis Moulos, Benjamin Breuil, Julie Klein, Nadia Lounis, Cécile Caubet, Stéphanie Tellier, Jean-Loup Bascands, Stéphane Decramer, Joost P. Schanstra, Bénédicte Buffin-Meyer

**Affiliations:** 1 Institut National de la Santé et de la Recherche Médicale (INSERM), U1048, Institut of Cardiovascular and Metabolic Disease, Equipe 12, 1 avenue Jean Poulhès, BP 84225, 31432 Toulouse Cedex 4, France; 2 Université Toulouse III Paul-Sabatier Toulouse, France; 3 HybridStat Predictive Analytics, Athens, Greece; 4 Unité de Recherche Clinique Pédiatrique, Module Plurithématique Pédiatrique, Centre d'Investigation Clinique - Hôpital des Enfants, Toulouse, France; 5 CHU Toulouse, Hôpital des Enfants, Service de Néphrologie – Médecine Interne – Hypertension Pédiatrique, Toulouse, France

## Abstract

Although capillary electrophoresis coupled to mass spectrometry (CE-MS) has potential application in the field of metabolite profiling, very few studies actually used CE-MS to identify clinically useful body fluid metabolites. Here we present an optimized CE-MS setup and analysis pipeline to reproducibly explore the metabolite content of urine. We show that the use of a beveled tip capillary improves the sensitivity of detection over a flat tip. We also present a novel normalization procedure based on the use of endogenous stable urinary metabolites identified in the combined metabolome of 75 different urine samples from healthy and diseased individuals. This method allows a highly reproducible comparison of the same sample analyzed nearly 130 times over a range of 4 years. To demonstrate the use of this pipeline in clinical research we compared the urinary metabolome of 34 newborns with ureteropelvic junction (UPJ) obstruction and 15 healthy newborns. We identified 32 features with differential urinary abundance. Combination of the 32 compounds in a SVM classifier predicted with 76% sensitivity and 86% specificity UPJ obstruction in a separate validation cohort of 24 individuals. Thus, this study demonstrates the feasibility to use CE-MS as a tool for the identification of clinically relevant urinary metabolites.

‘Omics’-based strategies appear to be promising tools for the identification of diagnostic and prognostic biomarkers of disease. They can lead to the design of multimarker models which are potentially better suited than single biomarkers to describe complex pathophysiological mechanisms^1^,^2^,^3^. Metabolomics, defined as the analysis of the low-molecular-weight compound (<1500 Da) content of a sample, offers advantages compared to the other omics traits. Indeed, being the downstream products of cellular function, metabolites represent a sensitive measure of the actions of upstream molecular species such as genes, transcripts, and enzymes, including the effects of disease, drugs, toxicity, and the environment^4^,^5^. However sensitivity to these many perturbants also contributes to potential issues about the high variability in metabolome exploration^6^.

Analysis of urine plays a central role in clinical diagnostics as it can be collected non-invasively, often in large quantities, and requires minimal sample pre-treatment due to its low complexity and protein content. In addition, we and others have already shown that urine is an excellent reservoir of biomarkers (peptides, proteins and metabolites) of many diseases^7^,^8^,^9^,^10^,^11^,^12^,^13^,^14^,^15^,^16^,^17^.

Metabolomics studies mostly use NMR spectroscopy and liquid chromatography coupled to mass spectrometry (LC-MS) that provide complementary readouts^4^. NMR spectroscopy allows both identification and quantification of metabolites. It is a highly reproducible and non-destructive method which requires minimal sample preparation thereby minimizing contamination and maintenance issues and enabling the routine and high-throughput analysis of hundreds to thousands of samples^4^,^18^. The inherent low sensitivity of NMR, however, restricts the detection limit to about 1 μM^18^,^19^. Moreover, the interpretation of NMR data is challenging^18^,^20^. In contrast, LC-MS allows the detection, quantification and structure elucidation of metabolites in the picomolar to nanomolar range of several thousand metabolites in a single measurement^5^. Unfortunately, the coupling of chromatographic separations with MS platforms requires an elevated level of maintenance, as the samples come in direct contact with many components of these platforms, contaminate surfaces and cause drift in the measured response and retention time over relatively short analysis periods^4^,^5^, thereby preventing the comparison of large numbers of samples. Relevant progress in the field of LC-MS was made with the introduction of ultra high performance liquid chromatography (UPLC) leading to improvement of analysis speed as well as sensitivity and resolution^19^,^21^,^22^. In particular, the potential of miniaturized UPLC-MS, based on the optimized use of microbore columns, was recently demonstrated for large-scale metabolomic studies^23^,^24^.

Until approximately ten years ago, capillary electrophoresis coupled to mass spectrometry (CE-MS) has only been rarely used for metabolome analysis. This was potentially due to issues related to stable coupling of CE to the MS instrument and the limited loading capacity of CE capillaries. However the significantly increased sensitivity of modern mass spectrometers and optimized methods for coupling of CE to MS have transformed CE-MS into a potential appropriate tool for profiling of disease associated metabolites in clinical relevant body fluid samples^20^,^25^,^26^,^27^,^28^,^29^. A number of recent studies now report the use of CE-MS for metabolome analysis of clinically relevant samples, with in particular those recently conducted by Soga and coworkers^30^,^31^,^32^, the first group to develop CE-MS for the comprehensive profiling of metabolites in biological samples^33^. However, the use of CE-MS for the discovery and validation of clinically relevant metabolic markers of human disease requires evaluation of its performance in terms of long term reproducibility and comparability.

Here, we present an optimized CE-MS setup and data analysis pipeline. Using a normalization procedure based on a set of “housekeeping” metabolites, this method allows to compare the metabolite content in urine samples analyzed over a period of several years. As proof of concept, we demonstrate the clinical relevance of this pipeline for the urinary metabolome based-detection of obstructive nephropathy in infants.

## Results

### Identification of metabolite internal standards for CE-MS normalization

**Figure 1:**
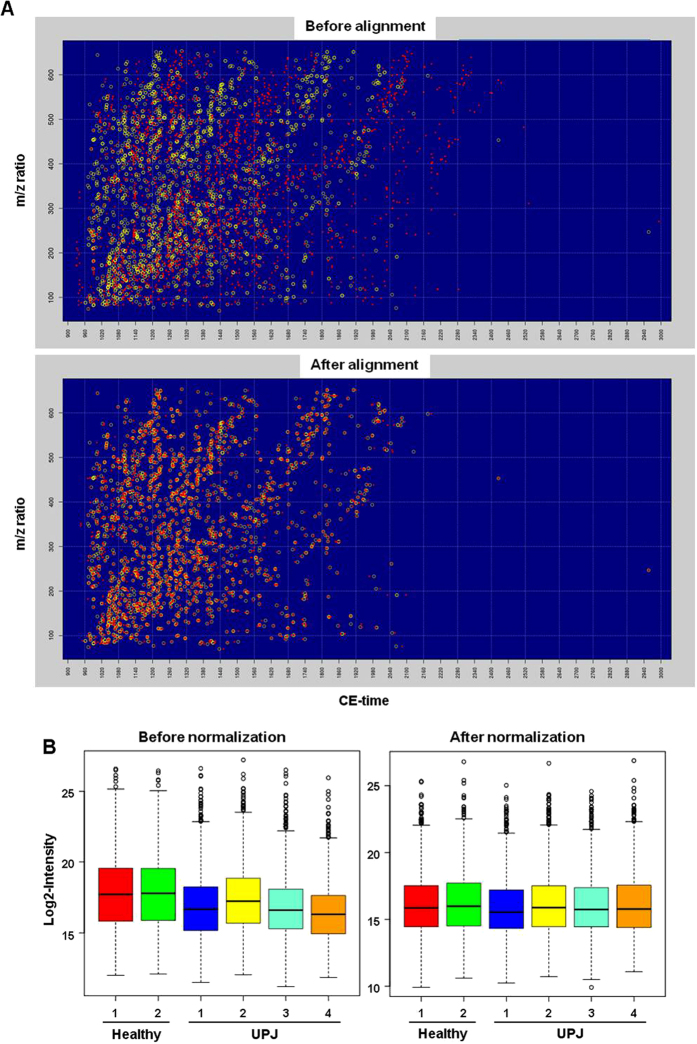
Processing and normalization of samples. Urinary samples were analyzed in CE-MS, processed and then normalized using the stable endogenous metabolites-based procedure described in the Materials and Methods section. (**A**) Representative distribution profile of urinary metabolite features before and after migration time alignment against reference dataset. Each circle is a unique peak processed with xcms. Red: metabolite features detected in a random urine sample and matching the reference; yellow: equivalent features in the reference dataset. (**B**) Box-whisker plot for metabolite abundance of exemplary healthy (2) and UPJ obstruction (4) patients before and after intensity normalization.

As a first measure towards improved comparison of large numbers of clinical samples over time, we developed a new method that allows to normalize the metabolite content of a biofluid sample. This method is based on the use of a set of persistent and stable metabolites across disease and healthy urine samples. In order to identify these so-called stable endogenous metabolites, 54 CE-MS runs of urine obtained from various kidney and urinary tract pathologies together with 21 control CE-MS runs of urine from healthy patients (Supplementary Table S1) were processed using the Bioconductor package xcms^34^. Each metabolite feature was identified by a unique identifier (ID) on the basis of the specific mass-to-charge ratio and migration time with a peak height representing the relative abundance. After preprocessing of the mass spectra (including mass calibration and migration time window restriction), the xcms pipeline (see Materials and Methods) identified 9642 distinct molecule features in terms of m/z and migration time pairs across all 75 samples. From this initial list, only features present (no-null abundance) in at least 50% of the total samples were considered for further analysis. The 6044 remaining metabolite features spanned a CE migration time from 16 to 50 min and a m/z range from 30–650. This reference dataset of 6044 metabolite features was then interrogated for the presence of stable molecule features, in terms of intensity, that would comprise the basis for a set of CE-MS internal normalization standards. For this, several established algorithms from the ‘rank invariant’ family of normalization methods present in the DNA microarray literature were deployed. Specifically, the Rank Invariant normalization method implemented in the dChip algorithm^35^, the Rank Invariant normalization algorithms for Illumina BeadArrays implemented in the lumi Bioconductor package^36^ and the GRSN algorithm^37^ were tested. However, each one of these suffered from several drawbacks, including among others unstable housekeeping sets because of their selection algorithm (dChip), selection preference in higher (dChip), lower (lumi) or medium (GRSN) intensities instead of spanning the whole metabolite abundance range, very high number of metabolites to achieve proper normalization (lumi) or poor normalization efficiency (dChip). The failure of present methodologies (partially due to the different nature of CE-MS data as compared to microarrays) to detect a stable set of metabolites led to the development of two new different internal standard selection strategies. Specifically, the first approach used the residuals of Robust Linear Regression models^38^,^39^ to identify sets of metabolites presenting low variability across samples and the second, more geometrical than statistical, approach was based on the Euclidean distance of each metabolite abundance vector from the identity ‘hyperline’ in the sample space. The final set of stable metabolites for each method was derived using a Forward Selection procedure with the purpose of finding the smallest possible subset of metabolites with the greater normalization power (detailed description of the methods in the ‘Materials and Methods’ section). The method that was finally followed was the geometrical approach as it was found to yield more robust results in terms of metabolite intensity coverage, normalization power, smaller number of stable metabolites and its application did not require any assumptions for a baseline as compared to the RLM approach which requires a baseline. This led to the identification of 267 endogenous housekeeping metabolic features among the 6044 features detected (Supplementary Table S2) which spanned a CE migration time from 17 to 36 min and a m/z range from 82 to 650. These stable endogenous metabolite features were implemented in the CE-MS normalization pipeline. Hence, the CE migration time is normalized in a first step (Fig. 1A) followed by normalization of the metabolite abundance using the endogenous housekeeping metabolic features, as exemplified on a random selection of six samples (Fig. 1B).

### Use of a beveled capillary improves the sensitivity of metabolite detection

CE coupling to MS via electrospray ionization (ESI) can be performed using either a sheathless or a sheath flow interface^40^. The use of sheathless systems is promising. In particular, the potential usefulness of a sheathless porous tip interface for CE-MS has been recently demonstrated for the analysis of the urinary metabolome^28^,^29^. Nevertheless this porous tip has not yet been adopted as a routine method for CE-MS coupling. So far, the sheath flow interface has been most widely used for CE-MS in metabolomics^26^,^40^,^41^. This type of coupling is stable and provides good sensitivity, its implementation is relatively easy and allows using a wide range of buffers. However, the CE-effluent is diluted in this configuration, thereby reducing the achievable sensitivity of the method^28^,^29^,^40^,^41^. As part of a continuous effort to improve the interface between CE and MS, Tseng *et al.*^41^ have developed a beveled tapered tip emitter in order to reduce the sheath flow leading to decreased sample dilution. By analyzing synthetic drugs and triazine mixtures, they demonstrated that the use of beveled tip provides better sensitivity for detection than conventional sheath liquid interface which uses flat capillary tips^41^.

**Figure 2:**
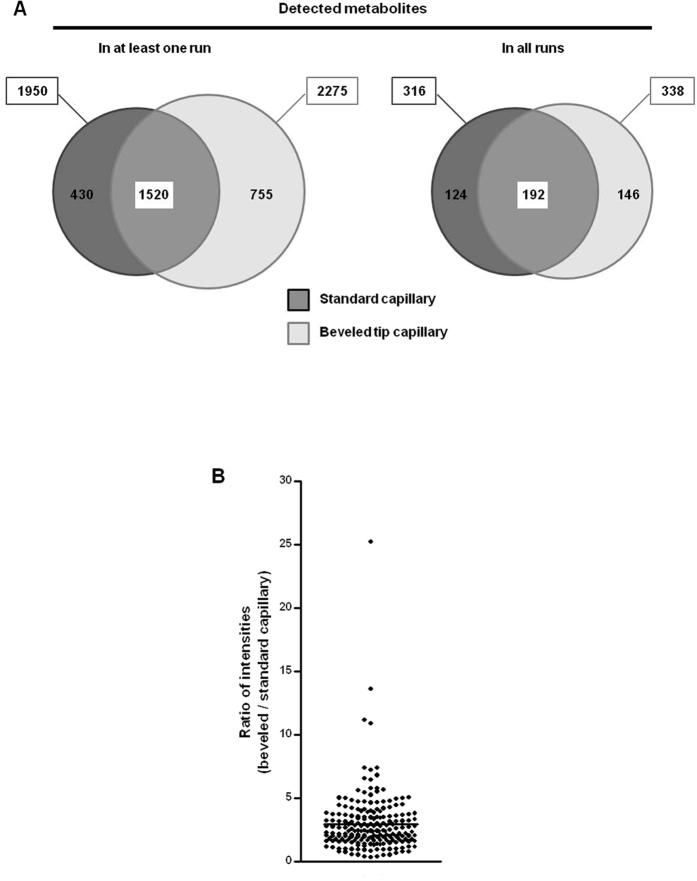
Effect of the capillary on sensitivity of metabolite detection. The same sample was analyzed in CE-MS using either standard (10 times) or beveled tip capillary (10 times) for CE. (**A**) Euler diagrams showing for each capillary the number of metabolite features detected at least once (left) or every time (right). Dark gray: standard (flat tip) capillary; light gray: beveled tip capillary. (**B**) For each metabolite detected in every run and with both types of capillaries (n = 192), the mean intensity was calculated and then the ratio between intensity measured with beveled tip capillary and intensity measured with classical capillary was calculated. Graph shows the mean ratio ± SEM, indicating that metabolite detection was more sensitive with beveled tip than with standard capillary.

Therefore in an attempt to optimize the sensitivity of the detection of urinary metabolites, we compared the performance of a standard flat tip and a beveled tip sheath-liquid ESI interface. A QC urine sample was analyzed by CE-MS using either a standard (ten consecutive runs) or beveled capillary (ten consecutive runs) for CE. Of note, the previously described 267 stable endogenous metabolites required for normalization procedure were identified using a beveled tip. After normalization, 2275 and 1950 distinct molecule features were detected in at least one run using the beveled tip and standard flat tip, respectively (Fig. 2A). Moreover, 338 and 316 metabolite features were detected consistently in all ten runs using the beveled tip and conventional tip, respectively (Fig. 2A). Although the absolute number of features detected is only slightly higher using the beveled tip, comparison of the intensities of 192 features detected in all runs with both types of capillary revealed a significant 3 fold gain in sensitivity using the modified capillary (Fig. 2B). Of note, robustness of the beveled tip was not decreased compared to flat tip (resisting to 40–50 runs [data not shown]). Therefore, the use of beveled tip as sheath-flow interface for CE-MS displays increased sensitivity towards the detection of urinary metabolites. We used the beveled tip for the remainder of the experiments.

### QC-based validation of CE-MS pipeline for urine metabolome profiling

In order to estimate the analytical variability of the CE-MS pipeline, a set of experiments for validation was performed: repeatability (intra-assay precision), postpreparation stability, postdilution stability, and long-term (intermediate) precision were evaluated.

**Figure 3:**
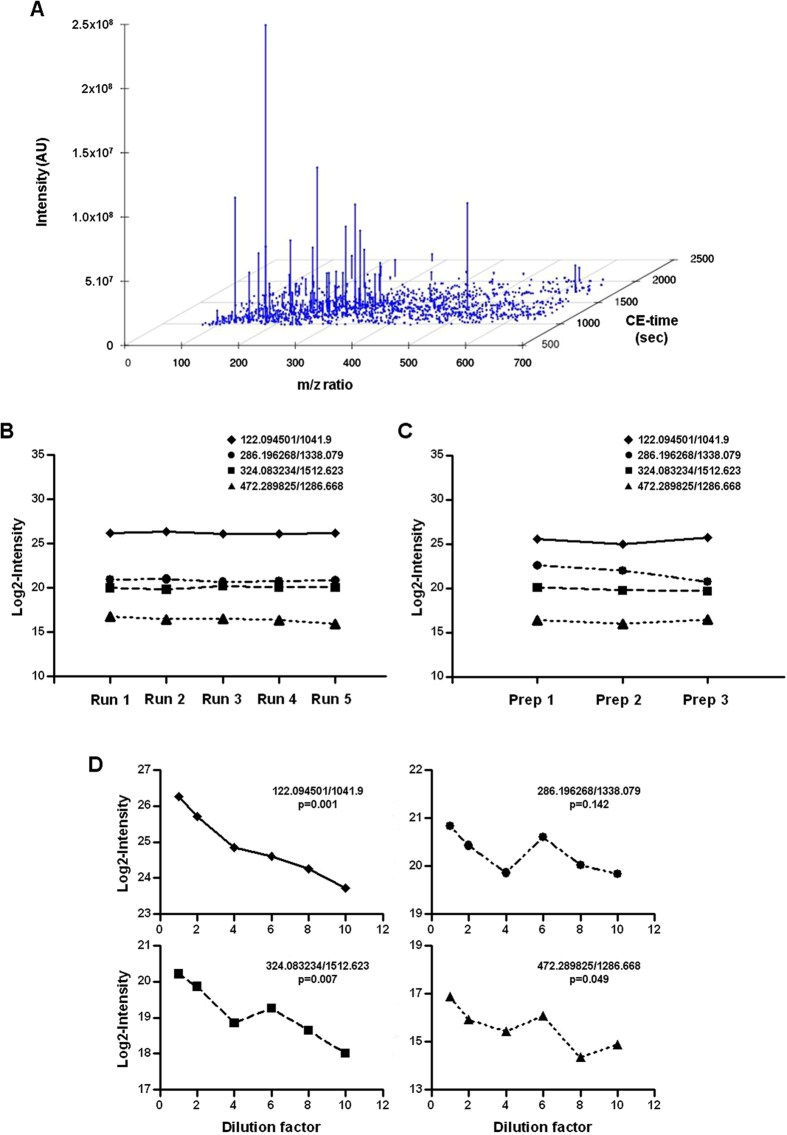
Short term performance characteristics of metabolomic CE-MS platform. The data from QC analyses were investigated to assess intra-assay precision, postpreparation stability and postdilution stability for molecule intensities. (**A**) Typical plot from the CE-MS analysis of the QC sample: Each metabolite was identified by a unique identifier (ID) on the basis of the specific mass-to-charge ratio and migration time. Graph shows the distribution of metabolite mass-to-charge ratio (*m*/*z*) with CE-migration time for a representative QC injection. (**B**) Short term precision: The QC was analyzed in five consecutive runs and the intensity in each run was shown for four exemplary randomly selected metabolite features. The coefficient of variance (CV) for amplitude was between 0.7 and 1.9% for these individual features, thereby demonstrating the repeatability in peak height. (**C**) Variability according to preparation: QC sample was prepared on three different dates using different lots of buffer, and then analyzed in consecutive runs. The intensity in each run was shown for four exemplary randomly selected metabolite features. The obtained CV for abundance was between 1.1 and 4.3%, showing a stability depending of the preparation. (**D**) Stability according to dilution: QC sample was prepared at different concentrations and then analyzed in consecutive runs. The intensity of four exemplary randomly selected metabolite features was plotted against the dilution factor.

Repeatability expresses the precision under the same operating conditions over a short interval of time. Repeatability of the CE-MS pipeline was examined by analyzing the QC urine sample in five consecutive runs, covering a total run time of ≈8 h. Among 6044 potential metabolites, 1342 (22%) features were detected on average in each run. Figure 3A shows a typical plot of a CE-MS analysis of a QC sample, giving an indication of the distribution of mass-to-charge ratio and CE migration times encountered for this typical sample. To obtain information on the run-to-run precision, four metabolite features were randomly selected for evaluation of intensity variation. The abundance variation of these four metabolite features was found to be negligible (Fig. 3B), with coefficient of variation (CV) values less than 2%, thereby indicating high performance of CE-MS platform in terms of repeatability. Next, the effect of different sample preparations was studied (post-preparation stability). We prepared QC sample according to the same procedure but using three different lots of buffer before CE-MS analysis in 3 consecutive runs. As shown in Fig. 3C, the intensity of the four exemplary selected metabolite features was constant in preparations, with a low CV, below 4.3%. Third, in order to test linearity of detection, the QC sample was prepared at six different concentrations and then analyzed by CE-MS in consecutive runs. Figure 3D depicts the abundance of the four randomly selected molecule features as a function of the dilution factor of a urine sample. For three of them, a significant negative correlation was observed between dilution and abundance whereas only a trend was observed for the fourth (Fig. 3D), thereby suggesting the relative stability of CE-MS platform when urine samples are diluted.

**Figure 4:**
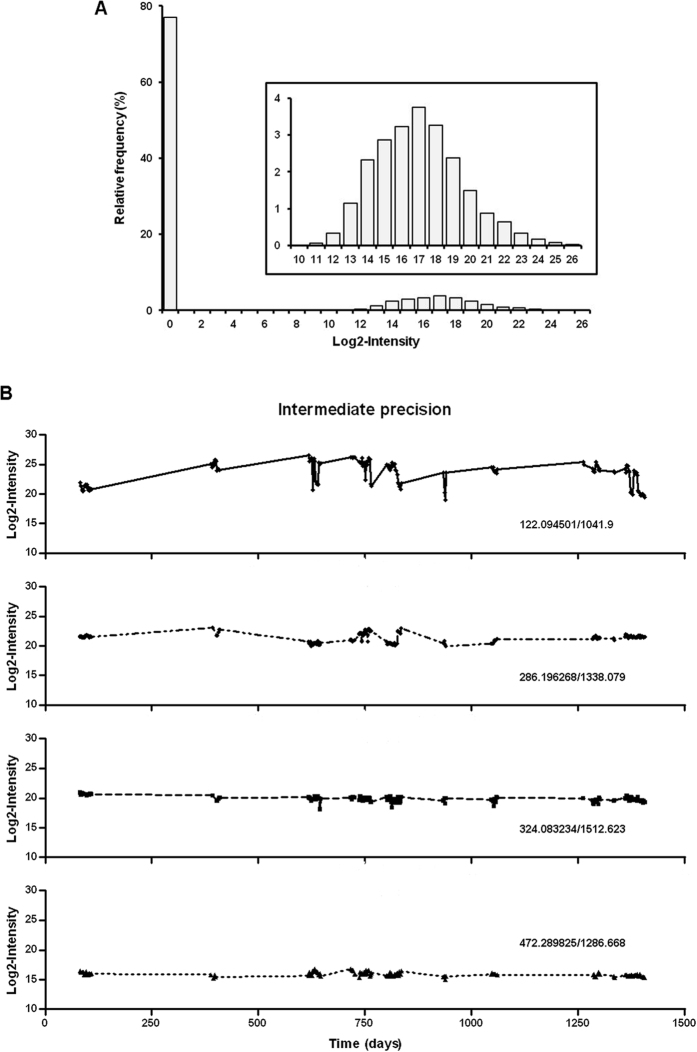
Long term performance characteristics of metabolomic CE-MS platform. The data from QC analyses were investigated to evaluate intermediate precision for molecule intensities. (**A**) Histograms of the distribution of abundance: The mean frequency of all features in QC sample was plotted against the logarithm (2) of the intensity. Profiles show a point-mass at zero and a continuous component. The zero component arises because the molecule features are either absent or their concentration is below the detection limit. Insert: magnification of the continuous distribution. (**B)** Long term variability: The QC sample was analyzed 128 times between 2011 and 2014. The intensity of four exemplary randomly selected metabolite features was plotted against the time.

Finally, we evaluated intermediate precision of CE-MS platform which expresses the precision within laboratory variations. This assay involved analysis of QC urine metabolites at different days by different operators over a long period of time. It included different lot numbers of buffers, solvents and chemicals and also implies annual maintenance service of both CE and MS devices. This evaluation is important in the field of clinically useful metabolite biomarkers where durable use of CE-MS is necessary. For the long-term stability assay, the QC sample was analyzed repeatedly 128 times over a range of 4 years (from 2011 to 2014). Among 6044 potential metabolite features, 1389 (23%) were detected on average in each run, this result being similar to the previously reported value. A mean of 67.7% of all metabolite features and 30.5% of the stable endogenous metabolites in the QC samples from these 128 runs matched against the reference dataset. The analysis of our data set revealed that the distribution of intensities is bimodal, with a strong proportion of values at a point-mass at zero (*point-of-mass values* [PMVs] corresponding to missing values [NaN], zero intensity data being treated as missing data) and a continuous component (Fig. 4A). The occurrence of zero component in the data matrix is a recurrent issue encountered in MS data^42^. The origin of PMVs may either be biological, eg absence of a specific metabolite in biological sample, or technical, eg the inability of the mass spectrometer to detect the specific metabolite or of the algorithm to identify the peak. Next, as it is recommended that the coefficient of variation should not exceed 15%^4^,^43^, we examined CE-MS results using similar acceptance criteria as a means of determining the quality of the data. For this, the abundance of four exemplary randomly chosen molecule features was plotted over time (Fig. 4B). The statistical spread for these metabolite features was between 2.2 and 8.6%, indicating that CE-MS platform exhibits long-term stability. In addition, CV of intensities was calculated for all metabolite features across the QC samples. A data subset was considered including features which were detected in at least one of the 128 QC injections (4879 entities) and different filters of selected metabolites were considered to evaluate improvement of the proportion of peaks being acceptable. Using this subset, we observed that 4487 (92%) of the 4879 molecule features displayed a variation of ≤10%, whilst 2892 (59%) exhibited a variation of ≤5% level. Altogether, these results demonstrated the long-term stability of CE-MS platform and thus suggest that the optimized CE-MS setup and analysis pipeline allows to compare the metabolite content in urine samples regardless of the time of analysis.

### CE-MS for clinical metabolomics: application to diagnosis of UPJ obstruction

Next we analyzed the capacity of the aforementioned pipeline in clinical research for the identification of diagnostic/prognostic biomarkers of disease. Newborns with UPJ obstruction were chosen for our proof of principle study. Two different cohorts of infants were employed: one discovery cohort (n = 49) for the identification of urinary metabolite biomarkers of UPJ obstruction (15 healthy newborns and 34 patients with UPJ obstruction; Table 1 and Supplementary Table S3) and one cohort (n = 24) for the blinded validation of urinary biomarkers (7 healthy newborns and 17 patients with UPJ obstruction; Table 2 and Supplementary Table S4). All urine samples were analyzed by CE-MS for their metabolite content and normalized using the above developed stable endogenous metabolites-based normalization procedure.

#### Metabolic profiling of urine samples from patients with UPJ obstruction and healthy children

**Figure 5:**
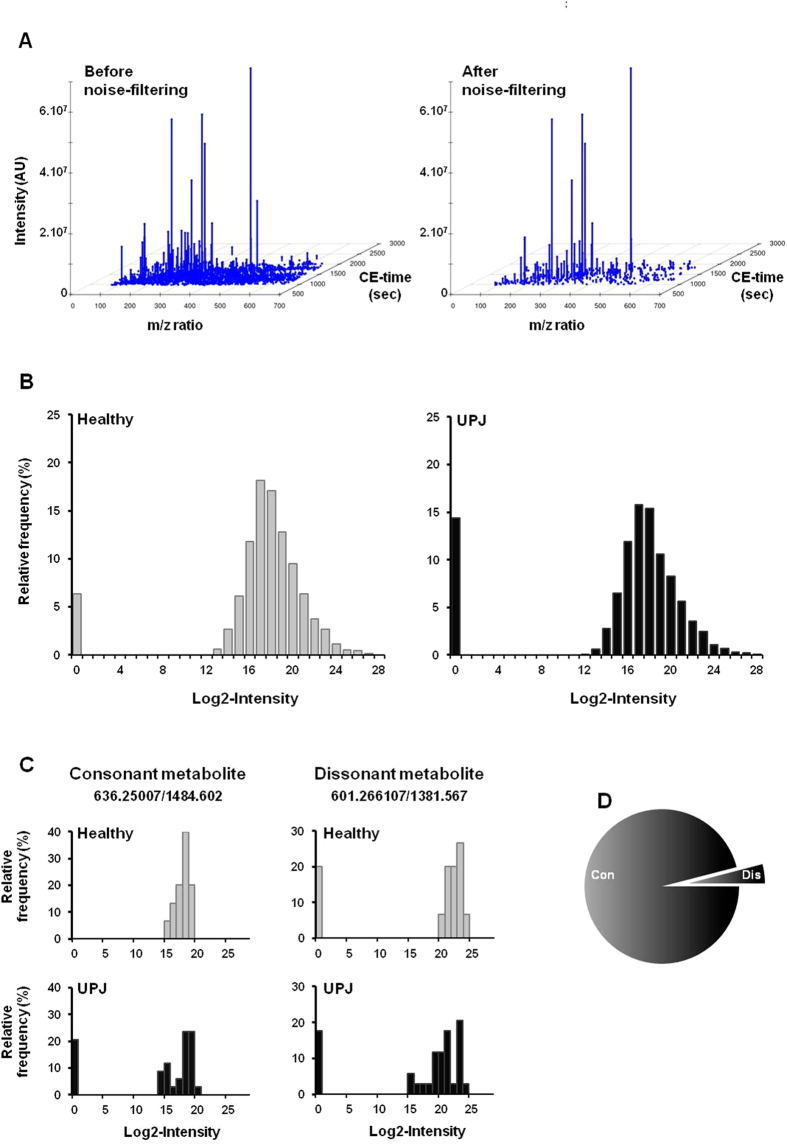
Metabolomic CE-MS analysis in urine of patients with UPJ. The urine metabolome of 15 healthy and 34 UPJ patients of the discovery cohort was analyzed. (**A**) Representative figure showing abundance of the CE-MS detected-urinary metabolite features: on the left, before application of a filter; on the right: after selection of features present in at least 75% of the samples in each group. (**B**) Histograms of distribution: The frequency of all metabolite features in healthy and UPJ samples was plotted against the logarithm (2) of the intensity. As for QC sample data, profiles show a point-mass at zero and a continuous component. (**C**) Histograms of distribution of two selected metabolite features from example dataset. Metabolite feature ID: 636.25007/1484.602 (left): consonant; metabolite feature ID: 601.266107/1381.567 (right): dissonant. (**D**) Repartition of compounds with consonant and dissonant differences between healthy and UPJ obstruction groups.

The urinary metabolome of the discovery cohort, composed of 15 healthy children and 34 patients with severe UPJ obstruction (Table 1 and Supplementary Table S3) was studied by CE-MS. A mean of 42.0% of the stable endogenous metabolites in urine samples matched against the reference dataset. Among 6044 potential metabolite features, 1889 (31%) were detected on average in each sample. Only the features detected in at least 75% of the urine samples in each group (healthy and UPJ) were further investigated. This noise-filtering process reduced the number of features to 388 entities (Fig. 5A). The distribution of the metabolite intensities for all the 388 selected metabolite features showed, as for QC sample data, a bimodal distribution characterized by a proportion of PMVs (Fig. 5B) and a continuous component. In order to explore the origin of PMVs, metabolite features with consonant or dissonant differences were quantified. In the former case, the group with the higher proportion of PMVs has the smaller mean in the continuous part, while in the latter case the group with the higher PMV proportion also has the higher mean. An example of each type is shown in Fig. 5C. Although this definition does not distinguish between technical and biological PMVs, technical PMVs naturally correspond to consonant compounds whereas biological PMVs generally allow for both types^44^,^45^. The data employed here contains 357 (92%) consonant compounds, 15 (4%) dissonant and 16 (4%) without point-mass component. The high proportion of consonant markers associated with the low number of dissonant markers suggests that PMVs in present metabolomics data originated from technical considerations rather than biological (Fig. 5D).

#### Identification of urinary metabolites associated to UPJ obstruction

**Figure 6:**
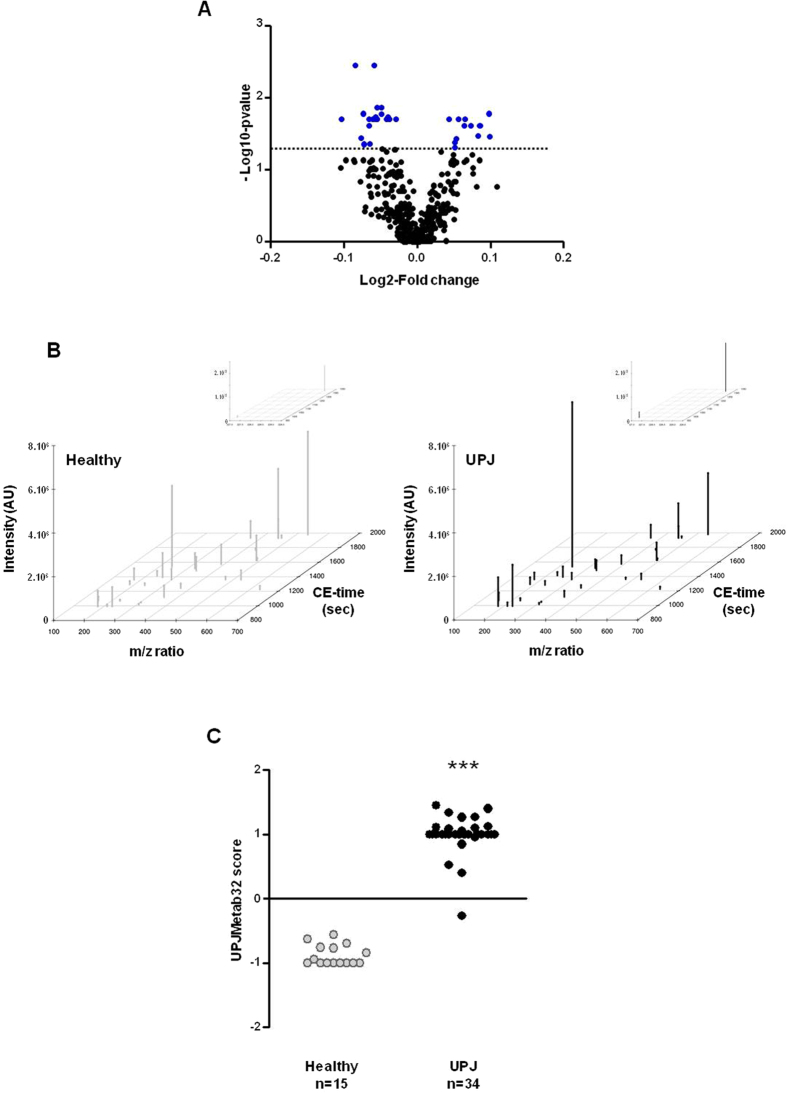
Identification of a classifier: UPJMeta32. The urine metabolome of 15 healthy and 34 UPJ patients (discovery cohort) was analyzed. (**A**) Volcano plot showing fold-changes (Log2) between UPJ obstruction and healthy groups as well as statistical significance (-Log 10 of p-value) for 388 considered metabolite features. The dashed line shows where p = 0.05. Points above the line had p < 0.05 and corresponding metabolite features (32) have been considered as significantly differentially excreted by UPJ patients. (**B**) Compared abundance of the 32 urine metabolite features which were identified as differentially excreted between UPJ patients and healthy subjects in the discovery cohort. Insert: two strongly abundant metabolite features (**C**) Cross-validation score of an SVM metabolite model, called UPJMetab32, consisting of 32 differentially excreted metabolite features. ***p < 0.0001 versus healthy subjects. Mann-Whitney test for independent samples.

Comparing urinary metabolites from UPJ and healthy patients led to the identification of 32 adjusted (Benjamini and Hochberg^46^) differentially excreted metabolite features (Fig. 6A,B and Supplementary Table S5). Matching 32 features against databases (HMDB, ChEBI and KEGG) led to determination of real mass for 9 metabolite features; 5 of 9 were annotated for chemical formulas (Table 3). Of note, abundances of two compounds (227.111791/989.758 and 228.114334/990.108) corresponding to the same annotation were highly correlated (R^2^ = 0.94, p < 0.0001, data not shown). The 32 metabolite features of interest were then used to develop a support vector machine (SVM) discrimination model that we called “UPJMetab32”. Scoring the patients from the discovery cohort with the UPJMetab32 classifier clearly separated UPJ from healthy patients (Fig. 6C).

#### Validation of UPJMeta32 in a separate, blinded cohort

**Figure 7:**
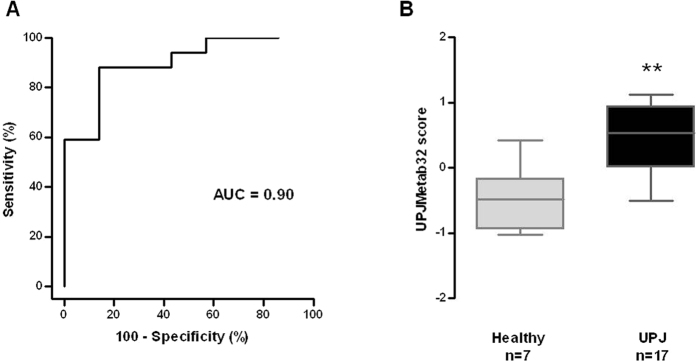
Validation of urinary metabolite classifier UPJMetab32 in a separate population. The diagnostic value of the UPJMetab32 model was tested in an independent cohort (7 healthy subjects and 17 UPJ patients) by a blinded analysis. (**A**) ROC curve for the UPJMetab32 classifier. (**B**) Box-whisker plot for classification of healthy and UPJ patients in the validation set according to the UPJMetab32 score. **p < 0.005 versus healthy subjects. Mann-Whitney test for independent samples.

In the next step, following the recommendations for biomarker identification^47^, the UPJMetab32 model was validated in a separate, blinded study using urine from 7 healthy and 17 UPJ patients not used in the discovery cohort (Table 2 and Supplementary Table S4). These urine samples were analyzed by CE-MS and scored using the UPJMetab32 model (Supplementary Table S4). A UPJMetab32 score >0 predicts patients with UPJ obstruction. These predictions were compared to the clinical criteria based status. The UPJMetab32 classifier diagnosed clinical status (healthy versus UPJ) with a sensitivity of 76.5%, a specificity of 85.7%, and an area under the curve (AUC) of 0.90 [95% CI: 0.707 to 0.984] (Fig. 7A). The UPJMetab32 model predicted 13 out of 17 UPJ cases correctly, showing the efficacy of the model to detect patients with severe UPJ. In addition, it predicted 6 out of 7 control cases correctly. The distribution of the UPJMetab32 scores for the validation cohort showed significant separation of the two patient populations (Fig. 7B).

## Discussion

We have explored the use of CE-MS and endogenous stable urinary metabolites for long-term, reproducible and comparable analysis of the urinary metabolome. The developed pipeline allowed comparison of urinary metabolite content analyzed over a 4 year timespan. As proof-of-concept we have used this pipeline to discover and validate urinary metabolites associated to a frequently encountered renal pathology in newborns.

Clinical metabolomics aims at the detection of clinically useful metabolites that can be extracted from a diverse range of sample types. Amongst those samples, easily accessible bodyfluids like urine and blood are most suited for clinical use. Although the field of metabolomics has advanced significantly in the past 10 years^4^, there has been little progress in the identification of clinically useful urinary metabolite biomarkers. To enable the discovery and the validation of diagnostic/predictive biomarkers, medium-to-large-scale epidemiological studies are required in order to take into account the substantial diversity observed in physiology/physiopathology, metabolic status and lifestyle in the general human population. This involves the use of analytical methods able to analyze large numbers of samples over periods of many months or years with both high reproducibility and high sensitivity^4^. We explored the potential of CE which offers multiple advantages: (i) as CE separates compounds on the basis of their charge and size^25^, it demonstrates high-resolution power for separation of small ionogenic metabolites which are important constituents of the urinary metabolome; (ii) CE separations require a low sample volume and consume very little solvent^25^, thereby reducing the matrix effect that can cause ion suppression and then insufficient ionization and lower peak intensity in MS; (iii) CE displays high reproducibility when analyzing large numbers of samples since no gradients are applied. Indeed, we observed high stability of urinary metabolite abundance when analyzing the same sample nearly 130 times over a range of 4 years. A few studies report stability evaluation of pipelines, such as for example over 535 runs covering a timespan of 5 months (GC-TOF-MS^48^) or over 120 runs covering a timespan of 3 years (UPLC-TOF-MS^49^). However, such a long term assessment of reproducibility and comparability is only rarely performed. Hence our 4 years proof of stability of the developed pipeline, associated with its use in the UPJ obstruction, validates its potential use in the clinic field.

Establishing long term stability has therefore been a major objective of the study. Although CE-MS is a reproducible analytical tool, some variations induced by sample concentration (especially for urine where individual urine outputs are dependent of water uptake, diet, …), interfering compounds and injection volume differences might still be observed. Several normalization strategies, such as normalization to creatinine, osmolarity and total area normalization are frequently employed in urine metabolomics studies. However, these commonly used normalization methods are not well adapted. For example, the creatinine level can be impacted by factors such as kidney function impairment, gender difference, and lean body mass^50^,^51^. The osmolarity normalization procedure is often affected by insoluble components, such as urine particles^50^,^52^. Adjusting the total peak area might yield biased results since the background noise and ion suppression due to the matrix may greatly interfere with the total signal. Furthermore, the total signal for samples with different metabolite distributions does not reflect the total concentration differences as ionization efficiency is compound dependent^50^. Variations can also be corrected by addition of exogenous standards but this method assumes that those are representative of the thousands of injected metabolites^53^. In the present study, we have opted for the selection of a set of most stable endogenous metabolites observed in a range of samples. This method offers several advantages. Firstly, for the selection of these stable endogenous compounds, we have chosen 75 urine samples potentially representing the diversity of (pediatric) diseases to be encountered in future studies. Therefore, we anticipate that the 267 derived stable endogenous metabolites can be used for the discovery of metabolite-based biomarkers in a number of pediatric diseases of the kidney and the urinary tract. Secondly, such a high number of stable endogenous metabolites for normalization spanning a CE migration time from 17 to 36 min and a m/z range from 82–650 allows that signal normalization can be performed ‘locally’, using metabolites with comparable ionization efficiency since close in terms of CE migration time and m/z ratio. In addition, this inclusion ensures that for every new sample, there will be a sufficient number of endogenous internal standards so as to span the whole intensity range of the new sample. Thirdly, as a result of this high number of stable endogenous metabolites, we observed that significant numbers of metabolite features are available for robust normalization in nearly all cases (we identified a mean of 42.0% of stable endogenous metabolites in the UPJ experiments). A potential drawback of the use of these endogenous metabolites for normalization could be that those are stable in the specific case of kidney disease and are excluded for the selection of biomarkers of disease. Selection of novel endogenous stable metabolites might thus be required in order to discover biomarkers for disease affecting other organs than the kidney/urinary tract.

Analysis of urinary metabolome is extremely attractive since changes reflect modifications of the entire organism in its equilibrium with the environment including particularly contributions from nutritive substances, drugs and gut microbial activities^19^. However, the variability induced by these factors can introduce a day-to-day intrapersonal variability as well as interpersonal differences, being a major drawback in studies aiming at disease diagnosis/prognosis. In order to address the sources of urinary metabolome variation throughout the day, Kim *et al.*^6^ have performed LC-MS metabolomics analysis of urine in subjects receiving a standardized and weight-based diet. The largest source of instability was attributable to technical issues such as sample preparation and analysis; to a lesser extent, an inconstancy subject-to subjects as well as intrapersonal variability due to meals and time of day were observed; day-to-day fluctuation was minimal^6^. Despite that, several studies suggest the existence of a stable part (time scale: months to years) of the urine metabolomic profile which seems to be specific to each individual^54^,^55^. Under unrestricted lifestyle conditions, multiple collections of urine samples can be used to reduce the metabolic noise and retrieve the individual phenotype^56^. In the current study, differences in alimentation are most likely not a confounding factor since alimentation of newborns/infants is significantly less variable than in adults.

We have show-cased the use of the pipeline in a frequently encountered renal pathology in newborns^11^,^57^. We were able to identify 32 metabolic features associated to UPJ obstruction. Combination of the 32 metabolite features in a SVM classifier predicted with 76% sensitivity and 86% specificity UPJ obstruction in a separate validation cohort, thereby demonstrating the efficacy of the model to detect patients with UPJ obstruction. Increased carnosine excretion in UPJ was attributed to two highly correlating isotopes of a same metabolite. Carnosine is a dipeptide synthesized from alanine and histidine by the carnosine synthase in muscle, brain and other tissues such as kidney. It is degraded by the carnosinase predominantly in the liver but also in kidneys. Carnosine from animal food can also be absorbed in the small intestine, and at least part of it enters the blood intactly upon oral ingestion. Finally, kidneys filter plasma carnosine, reabsorb a part of carnosine *via* specific transporters and excrete the remaining in urine^58^,^59^. In order to understand the origin of the elevated urinary level of carnosine from UPJ obstruction patients, further experiments measuring expression of carnosine related-enzymes and transporter proteins in both obstructed and contralateral kidneys should be performed. The dipeptide possesses also strong antioxidant and free radical scavenging activities^58^. Interestingly, protective effects of carnosine have been demonstrated in rodent models of kidney disease^60^,^61^,^62^ and in patients with diabetic nephropathy^63^ or children with glomerulopathies^64^. Thus, increased urinary excretion of carnosine in UPJ obstruction could be an adaptive rather than a deteriorating mechanism.

In conclusion, we have developed a robust setup and analysis pipeline for the exploration by CE-MS of the metabolite content of urine and found that the long-term reproducibility of the metabolite data generated was excellent. As proof of concept, we demonstrated the feasibility to use CE-MS as a tool for the identification of clinically relevant urinary metabolites.

## Materials and Methods

### Patients and urine collection

#### Samples used for optimization of the CE-MS normalization procedure

Fifty-four urinary samples from various kidney and urinary tract pathologies together with 21 control CE-MS samples from healthy patients (Supplementary Table S1) were used. We considered that these samples represent the potential diversity to be encountered in clinical samples and hence used those samples for the development of CE-MS normalization procedure.

#### Quality control (QC)

The QC sample was a mixture of urine samples of 9 healthy individuals (3 females and 6 males, mean age 34.1 ± 2.8 years).

#### Ureteropelvic junction (UPJ) obstruction and healthy patients

UPJ obstruction patients (n = 51) and healthy individuals (n = 22) of less than one year old were recruited in Toulouse Hospital and included in our study. The UPJ obstruction group was composed of patients scheduled for pyeloplasty with a pelvic dilatation of at least 16 mm and grade 3 and 4 hydronephrosis. Renographies were performed as soon as possible after birth, generally between week 3 and 6 to establish baseline differential renal function (DMSAscan) and washout pattern (MAG3-scan). Healthy and UPJ obstruction patients were randomly divided into two cohorts: a discovery cohort (n = 49; Table 1 and Supplementary Table S3) and a blinded cohort for validation (n = 24; Table 2 and Supplementary Table S4). Mann Whitney analysis revealed no significant difference in the age of healthy and UPJ obstruction newborns included in both discovery and validation cohorts (p = 0.26). In addition, the use of Chi Squared test also revealed no gender bias (p = 0.45). Urine from newborns was collected in the morning during 30 min using a sterile pediatric urine collection pouch (B. Braun, Boulogne, France) during hospital consultation. Urine from healthy controls was collected from newborns in the maternity hospital and at home using the same sterile collection bags and a pair of gloves. Care was taken to not take the first morning urine. After collection, all urines were frozen within the hour at −20 °C both in the hospital (dedicated −20 °C freezer in the clinic) and at home. Transport was done using ice blocks in both cases and the samples were finally stored at −80 °C in the laboratory. The UPJ study was performed in accordance with the ethical principles in the Declaration of Helsinki and Good Clinical Practice. The study and its experimental protocols were approved by the ethics committee of the French Ministry of National Education, Higher Education and Research (number DC-2008-452). Written informed consent was obtained from all participants (parents of the newborns).

### Sample Preparation

A 170 μl aliquot of urine was diluted with the same volume of a denaturing solution composed of 2 M urea, 0.0125% NH_4_OH, 100 mM NaCl and 0.01% SDS. To remove higher molecular mass proteins, the sample was ultrafiltered using a Centristat 20 kDa cut-off centrifugal filter device (Satorius, Göttingen, Germany) at 2000 × *g* for 45 min at 4 °C. In order to remove urea, electrolytes and SDS, 200 μl of filtrate was applied onto a NAP5 gel filtration column (GE Healthcare Bio Sciences, Uppsala, Sweden), washed and then eluted with 700 μl of 0.01% NH_4_OH. Finally, all samples were lyophilized in a Savant speedvac SVC100H connected to a Virtis 3L Sentry freeze dryer (Fischer Scientific, Illkirch, France). At this step, samples can be stored at 4 °C until use and re-suspended in HPLC grade water shortly before CE-MS analysis. The resuspension volume was adjusted to yield 1 μg/μl protein as measured by BCA assay (Pierce Biotechnology, Rockford, USA).

### CE-MS analysis

CE-MS analyses were performed as previously described^11^,^12^,^65^ using a Beckman Coulter Proteome Lab PA800 capillary electrophoresis system (Beckman Coulter, Fullerton, USA) on-line coupled to a micrOTOF II MS (Bruker Daltonic, Bremen, Germany). The electro-ionization sprayer (ESI, Agilent Technologies, Palo Alto, CA, USA) was grounded, and the ion spray interface potential was set between –4 and –4.5 kV. The CE separation buffer contained 20% (v/v) acetonitrile and 250 mM formic acid (Sigma-Aldrich) in HPLC-grade water. The CE-system was equipped with a 95 cm (internal diameter: 50 μm) bare fused silica capillary. Two types of CE-ESI-MS interfaces were tested (see results section); either a flatted or a tapered and beveled needle surrounding the capillary terminus. Data and MS acquisition methods were automatically controlled by the CE via contact-close-relays. Spectra were accumulated every 2 s, over a range of m/z 30 to 650.

### CE-MS sample preprocessing for stable endogenous metabolites identification

After mass calibration using the measurement of sodium formate salts at the start of each run, the raw MS-data were converted into NetCDF format (http://www.unidata.ucar.edu/software/netcdf/) through the Bruker software (DataAnalysis version 4.0). The NetCDF files were filtered by excluding spectra corresponding to a migration time less than 520 or greater than 3650 seconds prior to preprocessing using the Bioconductor package *xcms*^34^ as previously described^25^. All the standard xcms pipeline parameters were kept to their defaults apart from *steps* which was set to 3 and *bw* which was set to 20. In addition, the total number of migration time alignment iterations was set to 5, using the LOESS approach of xcms. The resulting molecule features derived from the execution of the xcms pipeline (in terms of m/z and migration time pairs) were further filtered for their presence across samples by including only those molecule features present in at least 50% of the total samples. The latter ensured the robustness of the initial set of molecule intensities which would be later interrogated for the presence of stable (in terms of intensity) molecule features that would serve as a set of CE-MS internal normalization standards.

### Stable endogenous metabolites identification

The final filtered set of xcms preprocessed and identified m/z – migration time pairs was further interrogated for the potential presence of a set of ‘housekeeping’ metabolites with stable intensity across pathologies and spanning the whole intensity range. To this end, a subset of ‘rank invariant’ family of normalization algorithms from the DNA microarray literature was applied with the purpose of identifying stable molecule features that would represent the ‘invariant set’ as referenced in the microarray bibliography^66^. Specifically, the algorithms described in^35^ (*dChip* algorithm)^36^, (*lumi* Bioconductor package) and^37^ (*GRSN* algorithm) were applied and sets of rank invariant metabolite abundances were retrieved. However, graphical assessment of the performance of these algorithms (see main text) revealed that the nature of CE-MS data prohibited the usage of these algorithms for the identification of a set of internal standards. Therefore, the following two strategies were applied:

1. The first strategy is based on the assumption that the majority of identified metabolites do not present differential abundance across samples (a similar assumption made for the normalization methods in the DNA microarray literature) and as a result, the relationship among different sample abundances is close to linear, after xcms preprocessing. Specifically, this approach includes the fitting of a set of Robust Linear Regression models^38^,^39^, either among all possible sample pairs, or for all samples against a baseline (e.g. the median metabolite abundances across samples) and the calculation of each model residuals. The set of stable metabolites is iteratively constructed by aggregating those ones whose abundance presented very low residuals in each model, implying low divergence from the model and subsequently, among samples.
2. The second strategy does not make any assumptions about the differential abundance distribution of the metabolites but requires noise preprocessing, as performed by the xcms pipeline and is based on the geometrical distance of each metabolite abundance vector in the sample space from the identity ‘hyperline’. Specifically, this approach includes the construction of the identity ‘hyperline’ 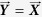, in the *n*-dimensional sample space, where 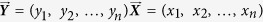, and *n* is the number of samples. Then for each metabolite abundance vector 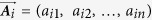, the Euclidean distance 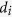 from an equally spaced grid distributed along 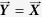 is calculated. The set of stable metabolites is constructed by aggregating metabolites with small *d*_*i*_ which imply both very high correlation as well as low inter-sample variability.

In both strategies (i) and (ii), the optimal number of metabolites with stable abundances is selected according to the normalizing potential of forward selected subsets of metabolites. The Forward Selection approach was selected as the number of stable metabolites should be kept to the minimum possible also for later purposes (exploration of prognostic or diagnostic values). Specifically, the initial candidate list is constructed by retrieving the first 1000 metabolites with the smallest Euclidean distance from the identity hyperline (or with the smallest residual value from an RLM) and sorting it in ascending order (of distance or residual value). Then, starting from a minimum set *S* of 10 metabolites, the whole dataset is normalized by fitting a LOESS curve *L* in this set and using it as the normalization reference. In each iteration one member of the stable metabolite candidate list is added to *S*, *L* is recalculated, the whole dataset is normalized and the following dataset variability metric is calculated:


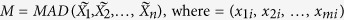


and *x*_*ij*_ the normalized abundance of metabolite *i* in sample *j*, *i* = *1, …, m (m* the number of metabolites in the total dataset), *j* = *1, …, n (n* the number of CE-MS samples). *M* reflects the total variability of the normalized intensity matrix by firstly summarizing each column (sample) by taking its median value and then calculating the variability of this summarization, by taking the Median Absolute Deviation (MAD) of the column medians distribution. The final number of the stable metabolites is the size of *S* that minimizes *M* and has thus the best normalizing potential while at the same time being as small as possible.

### Processing and normalization of new samples

New CE-MS urine samples are preprocessed up to filtering (exclusion of spectra corresponding to a CE-time less than on average 840 [sodium salts] or greater than 3000 seconds) and peak-picking (no migration time alignment) with xcms as described above. Then, the masses of the new samples are matched against the reference dataset (consisting of 75 disease and control runs as described above) with a tolerance of 0.01 mass units, and the molecule features that do not match the reference are excluded from further analysis. The migration time alignment of the new samples is performed with an iterative procedure, similarly to the one followed by the xcms package but using the urine specific internal standards instead of the ones that are identified for independent datasets by xcms. Specifically, the migration times of the internal standards subset which is specific to the new sample (identified as described above) and span the whole range of the new sample’s migration times, are used as seeds for the creation of migration time clusters using *k-means* clustering with the *k* parameter equal to the number of matching internal standards. Then, a LOESS curve is fitted to each cluster and used as a reference for the alignment of migration times in each cluster. The intensity normalization of the new samples is performed as described above (‘stable endogenous metabolites identification’ section), using the proper subset of the internal standards set according to the aforementioned mass match procedure.

### Metabolite features annotation

The final set was matched against HMDB^67^, ChEBI^68^ and KEGG^69^ for known molecules and annotated for potential chemical formulas using the *CAMERA* Bioconductor package^70^. From the two aforementioned methods, RLM and identity hyperline, the latter was selected as it was found to yield more robust results in terms of metabolite intensity coverage (*S* contained features spanning a sufficient range of intensities), normalization power, cardinality of *S* and its application did not require any assumptions for a baseline.

### Statistical analysis

#### Biomarker identification and modelling

For the identification of potential metabolite biomarkers, the normalized levels of urinary metabolite features were compared between the healthy and UPJ obstruction patient groups. Only molecule features that were detected with a minimal frequency of 75% in every of the discovery groups were investigated for statistical analysis. Missing values (recorded as “Not a Number” [NaN]) from the discovery cohort were replaced by the average of the metabolite intensities found in the corresponding group (UPJ obstruction patients or healthy newborns). However, in the validation cohort where the belonging of the sample is unknown, we used the mean abundance of all patients from discovery set as imputation methods for missing values. Of note, zero values were considered as missing values. P-values were calculated for the comparison between healthy and UPJ obstruction patient groups using the Wilcoxon test followed by adjustment for multiple testing using the method described by Benjamini and Hochberg^46^. Only metabolite features with a corrected p < 0.05 were considered significant. Using an in-house developed tool, we next used a support vector machine (SVM)-based approach (SVM package e1071 of R)^71^ to generate a prognostic biomarker classifier based on 32 biomarkers associated with UPJ obstruction. The parameters of the radial kernel function (type C) for the multi-dimensional hyperplane were: cost parameter (C) of 1 and kernel width (γ) of 0.03125. Sensitivity and specificity were calculated using receiver operating characteristic (ROC) plots *via* the software R.

#### Comparison of svm scores

Statistical analyses were performed using GraphPad Prism 5.0 for Windows (GraphPad Software Inc) and comparisons between two groups were assessed using a Mann-Whitney test for independent samples. p < 0.05 was considered as statistically significant.

## Additional Information

**How to cite this article**: Boizard, F. *et al.* A capillary electrophoresis coupled to mass spectrometry pipeline for long term comparable assessment of the urinary metabolome. *Sci. Rep.* **6**, 34453; doi: 10.1038/srep34453 (2016).

## Supplementary Material

Supplementary Information

## Figures and Tables

**Table 1:** Discovery cohort.

	All patients	Healthy	UPJ obstruction
n	49	15	34
Gender			
M	46 (93.9%)	15 (100%)	31 (91.2%)
F	3 (6.1%)		3 (8.8%)
Age			
Mean (months)	2.25 +/− 0.27	2.29 +/− 0.62	2.22 +/− 0.29
Median (months)	1.45 (range 0 to 7.0)	1.58 (range 0 to 6.1)	1.45 (range 0.7 to 7.0)

**Table 2:** Validation cohort.

	All patients	Healthy	UPJ obstruction
n	24	7	17
Gender			
M	21 (87.5%)	7 (100%)	14 (82.4%)
F	3 (12.5%)	0	3 (17.6%)
Age			
Mean (months)	2.51 +/− 0.58	1.35 +/− 1.21	2.99 +/− 0.65
Median (months)	1.28 (range 0 to 8.6)	0.03 (range 0 to 8.6)	1.61 (range 0.8 to 8.6)

**Table 3:** Annotation for potential chemical formulas and names.

ID	Isotope	Adduct	Real Mass	Database	Database code	Proposed Formula	Proposed Name
227.111791/989.758	[56][M]+	[M+H]+	226.104515	HMDB	HMDB00033	C9H14N4O3	Carnosine
228.114334/990.108	[56][M+1]+			KEGG	cpd:C00386	C9H14N4O3	Carnosine
				HMDB	HMDB12482	C9H14N4O3	Hydroxypterin
				HMDB	HMDB00245	C10H14N2O4	Porphobilinogen
				KEGG	cpd:C00931	C10H14N2O4	Porphobilinogen
				KEGG	cpd:C02345	C15H14O2	(2S)-Flavan-4-ol
				KEGG	cpd:C15598	C15H14O2	Favan-3-ol
				KEGG	cpd:C09757	C15H14O2	7-Hydroxyflavan
				KEGG	cpd:C10276	C15H14O2	Pinosylvinmethylether
				KEGG	cpd:C10325	C15H14O2	Deoxylapachol
				KEGG	cpd:C13632	C15H14O2	4,4′-Dihydroxy-alpha-methylstilbene
				KEGG	cpd:C07205	C14H14N2O	Metyrapone
				ChEBI	55316	C7H16BrNO2	Acetylcholine bromide
				ChEBI	50426	H4O6P2S2	Disulfanediylbis(phosphonic acid)
229.117309/1322.695	[8][M]+	[M+H]+	228.110033	HMDB	HMDB06695	C10H16N2O4	Prolylhydroxyproline
				KEGG	cpd:C13733	C10H16N2O4	(S)-ATPA
				KEGG	cpd:C10371	C15H16O2	MansononeC
				KEGG	cpd:C13624	C15H16O2	BisphenolA
				KEGG	cpd:C15210	C15H16O2	1,1-Bis(4-hydroxyphenyl)propane
				KEGG	cpd:C17424	C15H16O2	Lindenenone
				ChEBI	58089	C5H11NO7P	5-phosphonato-D-ribosylaminium(1−)
				ChEBI	58681	C5H11NO7P	5-phospho-β-D-ribosylaminium(1−)
				KEGG	cpd:C18436	C9H16N4OS	Tebuthiuron
				ChEBI	53648	C7H4N2O7	2-hydroxy-3,5-dinitrobenzoic acid
355.071351/1117.064	[306][M]+		354.064075	KEGG	cpd:C01268	C9H15N4O9P	5-Amino-6-(5′-phosphoribosylamino)uracil
				KEGG	cpd:C02927	C15H14O10	2-Caffeoylisocitrate
				KEGG	cpd:C07952	C17H19ClN2S. HCl	Chloropromazinemonohydrochloride
				KEGG	cpd:C12600	C19H14O5S	Phenolsulfonphthalein
488.133087/1622.375	[453][M]+		487.125811	KEGG	cpd:C02555	C26H21N3O5S	Luciferylsulfate
				KEGG	cpd:C18429	C18H22FN5O8S	Flucetosulfuron
366.599792/1929.853	[461][M]+		365.592516				
438.677677/1763.369	[443][M]+		437.670401				
474.701959/1359.882	[359][M]+		473.694683				
526.161131/1357.627	[160][M]+		525.153855				

HMDB: Human Metabolome Data Base; KEGG: Kyoto Encyclopedia of Genes and Genomes, ChEBI: Chemical Entities of Biological Interest.
