# Supplementary material for: A capillary electrophoresis coupled to mass spectrometry pipeline for long term comparable assessment of the urinary metabolome
Source: Sci Rep. 2016 Oct 3;6:34453. doi: 10.1038/srep34453 (PMC5046087; doi:10.1038/srep34453)
Supplement: Supplementary Information [file srep34453-s1.pdf]

Supplementary Information

A capillary electrophoresis coupled to mass spectrometry pipeline for long term comparable assessment of the urinary metabolome

Franck Boizard[1,2,#], Valérie Brunchault[1,2,#], Panagiotis Moulos[3], Benjamin Breuil[1,2], Julie Klein[1,2], Nadia Lounis[4], Cécile Caubet[1,2], Stéphanie Tellier[5], Jean-Loup Bascands[1,2], Stéphane Decramer[1,2,5], Joost P Schanstra[1,2*], Bénédicte Buffin-Meyer[1,2*]

[1] Institut National de la Santé et de la Recherche Médicale (INSERM), U1048, Institut of Cardiovascular and Metabolic Disease.

[2] Université Toulouse III Paul-Sabatier Toulouse, France.

[3] HybridStat Predictive Analytics, Athens, Greece.

[4] Unité de Recherche Clinique Pédiatrique, Module Plurithématique Pédiatrique, Centre d'Investigation Clinique - Hôpital des Enfants, Toulouse, France

[5] CHU Toulouse, Hôpital des Enfants, Service de Néphrologie – Médecine Interne – Hypertension Pédiatrique, Toulouse, France.

[#] Franck Boizard and Valérie Brunchault contributed equally to this work.

* Co-correspondence to:
joost-peter.schanstra@inserm.fr and benedicte.buffin-meyer@inserm.fr

Supplementary Table S1: Characteristics of the cohort used for identification of the endogenous housekeeping metabolic features.

**Gender**		
M	46	61.3%
F	29	38.7%
**Clinic symptoms of renal pathology**		
no renal pathology	21	28.0%
ureteropelvic junction obstruction	25	33.3%
pelvicalyceal dilatation	4	5.3%
posterior urethral valves	4	5.3%
megaureter	3	4.0%
pyelonephritis	3	4.0%
hyperechogen kidney	3	4.0%
ureteral hydronephrosis	3	4.0%
bilateral renal hypoplasia	2	2.7%
multicystic kidney	2	2.7%
unique kidney	2	2.7%
renal cystic dysplasia	1	1.3%
glomerulonephritis	1	1.3%
maternity	1	1.3%

Supplementary Table S2: List of endogenous housekeeping metabolic features

ID	mass-to-charge ratio	CE-time (min)	Log2-intensity
82.064873/1368.589	82,06487274	22,8098192	14,68
100.074386/1290.237	100,0743857	21,50395422	21,50
126.090263/1290.666	126,0902629	21,51110489	19,34
131.04454/1408.987	131,0445404	23,48310929	19,25
134.043624/1361.785	134,0436241	22,69641784	20,62
152.579489/1215.841	152,5794887	20,26401059	13,50
164.107226/1230.268	164,1072264	20,50447002	18,05
164.770715/1144.141	164,7707148	19,06901749	12,66
165.537382/1144.661	165,5373824	19,07768342	12,69
166.597455/1194.84	166,5974554	19,914004	13,16
179.098782/1060.965	179,0987821	17,68274446	19,37
191.064945/1361.393	191,064945	22,68987572	20,19
198.601243/1201.964	198,6012432	20,03273491	13,73
202.500848/1344.512	202,5008476	22,4085376	13,52
203.136101/1300.548	203,1361008	21,67580248	23,07
204.14024/1296.284	204,1402399	21,60474124	20,45
212.615842/1208.194	212,6158423	20,13656831	13,98
222.60553/1222.569	222,6055304	20,37615281	13,36
228.589951/1160.622	228,5899513	19,34369431	13,66
231.607345/1221.787	231,6073454	20,36311795	14,50
235.604147/1214.61	235,6041466	20,24350635	13,78
236.611114/1286.885	236,6111144	21,44807627	13,69
246.611275/1469.58	246,6112747	24,49299902	13,87
250.61946/1288.443	250,6194598	21,47405246	13,33
254.612435/1213.68	254,612435	20,22800526	13,78
255.622652/1222.513	255,6226521	20,37522417	13,07
256.614786/1262.341	256,6147862	21,03901713	14,60
260.189836/1017.354	260,1898359	16,95589886	21,14
260.740627/1465.128	260,7406272	24,41880129	14,03
261.25445/1017.45	261,2544502	16,95750329	15,26
262.112181/1421.234	262,1121809	23,68723898	23,07
265.633773/1179.398	265,6337732	19,65664051	14,62
268.045356/1743.858	268,0453562	29,0642968	16,60
268.622288/1258.926	268,6222881	20,9820975	13,79
269.635473/1230.377	269,6354726	20,50627711	14,72
270.6254/1212.424	270,6253996	20,20706497	13,98
270.63942/1359.559	270,63942	22,65931457	13,73
271.636821/1202.838	271,6368213	20,04730355	16,10
273.628936/1194.162	273,6289358	19,90270664	14,30
274.629864/1191.02	274,6298635	19,85034113	14,13
274.630437/1262.888	274,6304374	21,0481343	14,41
281.143806/1204.434	281,1438063	20,07390411	19,58
282.665381/1153.988	282,665381	19,23313141	13,84
285.575663/1815.207	285,5756634	30,25345542	13,36
285.612072/1263.691	285,6120721	21,0615105	14,13

289.616079/1364.636	289,6160794	22,74393441	14,04
289.633751/1214.821	289,6337508	20,24700843	15,15
290.636694/1206.118	290,6366936	20,10197092	14,74
292.621738/1205.03	292,621738	20,08383204	16,21
294.630044/1199.84	294,6300443	19,9973258	15,79
294.64226/1362.038	294,6422602	22,70063152	13,41
296.654961/1271.452	296,654961	21,19086441	14,91
298.631463/1220.712	298,6314635	20,34519762	14,37
298.635515/1293.916	298,6355149	21,56527263	14,62
299.646088/1197.092	299,6460876	19,95153484	15,99
300.590944/1408.706	300,5909444	23,47843393	13,71
300.650874/1217.639	300,6508739	20,29397918	17,35
302.133273/1411.849	302,1332732	23,53082121	23,15
302.135969/1287.865	302,135969	21,46440995	20,12
303.13451/1413.158	303,1345101	23,55262564	20,95
304.111015/1593.291	304,1110154	26,55484677	21,92
304.648005/1268.013	304,6480047	21,13354378	16,32
305.63435/1302.773	305,6343504	21,71287654	15,12
307.146323/1369.066	307,1463235	22,817772	18,62
307.148705/1230.369	307,1487049	20,50614996	19,07
308.665297/1169.656	308,6652966	19,49426678	14,04
310.638959/1255.701	310,6389587	20,92835776	13,79
314.101344/1743.897	314,1013436	29,0649441	18,79
314.634715/1251.179	314,6347146	20,85298135	15,97
318.643809/1223.993	318,6438086	20,39988076	14,52
319.159081/1102.727	319,1590809	18,37878525	19,39
320.13298/1485.611	320,1329803	24,7601903	20,37
320.635477/1213.982	320,6354768	20,2330331	15,35
321.641887/1305.62	321,6418868	21,76034121	15,11
321.650428/1208.409	321,6504277	20,14014278	15,39
323.630609/1801.983	323,6306086	30,03305525	13,83
323.640607/1249.625	323,6406065	20,82708473	16,90
324.150607/1248.284	324,150607	20,8047335	16,67
324.647138/1249.004	324,6471381	20,81674097	15,53
325.649757/1288.357	325,6497575	21,47261704	14,11
327.161675/1369.801	327,1616752	22,83001681	17,43
327.162473/1230.925	327,1624728	20,51541556	19,53
328.124035/1141.391	328,1240353	19,0231778	19,09
328.671614/1184.054	328,6716143	19,73424161	14,87
331.644158/1207.185	331,6441578	20,11974746	15,90
333.646652/1259.013	333,6466523	20,98354474	13,84
335.15515/1273.104	335,1551495	21,21840159	16,63
337.653498/1390.386	337,653498	23,17309764	13,95
342.135439/1743.897	342,1354392	29,0649441	19,19
343.661957/1224.444	343,6619573	20,40740632	15,98
348.147758/1301.265	348,1477582	21,68775823	21,03
348.64621/1326.937	348,6462097	22,11562184	14,59
349.150317/1301.565	349,1503175	21,69275782	18,52
351.687587/1345.166	351,687587	22,41942605	14,58
352.060866/1538.349	352,060866	25,63914717	17,44
352.696232/1254.963	352,6962316	20,9160515	16,68
353.636588/1256.701	353,6365877	20,9450182	14,62

353.655923/1336.987	353,6559232	22,28311962	14,00
356.043978/1484.516	356,0439784	24,74192537	16,78
357.240469/1045.095	357,2404687	17,41825461	19,67
357.668794/1243.614	357,6687942	20,72690523	15,21
358.218428/1045.336	358,2184278	17,42227492	18,22
360.652745/1399.776	360,6527447	23,3296071	13,83
360.661529/1303.782	360,661529	21,72970736	14,15
361.718911/1215.219	361,7189114	20,25364527	14,91
365.654915/1256.415	365,6549154	20,94025609	16,08
366.599792/1929.853	366,5997925	32,16421764	18,61
368.654864/1319.063	368,6548638	21,9843821	14,65
369.66691/1355.09	369,6669101	22,58483945	13,91
370.676917/1353.474	370,676917	22,55789869	14,67
373.168936/1550.871	373,168936	25,84785716	19,76
375.22096/1068.053	375,2209604	17,80087744	21,27
375.645865/1258.961	375,6458654	20,98268539	13,77
376.218952/1070.119	376,2189515	17,83531475	19,05
377.16708/1163.562	377,1670795	19,39269307	17,61
377.655953/1359.433	377,6559532	22,65721418	15,56
378.110994/1784.113	378,1109936	29,73521871	18,01
378.659033/1291.424	378,6590332	21,52372702	17,01
379.671845/1355.508	379,6718446	22,59179673	14,88
381.688107/1297.209	381,6881069	21,62015418	15,24
385.166963/1664.281	385,1669625	27,73802389	18,91
385.173767/1266.758	385,1737671	21,11262804	20,18
385.648308/1368.425	385,6483075	22,80709165	14,80
387.174605/1288.03	387,1746047	21,46716792	19,04
389.197597/1336.085	389,1975971	22,26808683	17,09
391.178399/1158.617	391,1783986	19,31029045	17,20
392.18842/1099.733	392,1884198	18,32888225	17,71
392.637684/1364.239	392,6376836	22,73731927	14,12
395.127839/1864.154	395,1278386	31,06923162	16,68
395.629514/1865.679	395,6295141	31,09464439	14,84
397.678509/1356.868	397,6785088	22,61446805	14,82
398.676458/1217.373	398,676458	20,28954232	14,60
401.165412/1630.778	401,1654115	27,17962894	17,16
401.172738/1694.078	401,1727382	28,23463127	17,85
405.160292/1750.745	405,1602925	29,17908622	17,69
407.681908/1288.138	407,6819084	21,46896308	15,95
408.196932/1586.633	408,1969318	26,44388156	18,85
410.173096/1595.747	410,1730957	26,59578908	19,37
413.200371/1192.737	413,2003708	19,87894394	18,29
413.683915/1159.411	413,6839146	19,32352332	15,16
416.138318/1457.816	416,1383182	24,29692872	21,18
417.138051/1457.816	417,1380508	24,29692872	19,16
422.208861/1585.041	422,208861	26,41734307	19,27
423.21461/1582.481	423,2146104	26,37469114	17,03
428.171946/2161.302	428,1719461	36,02170451	19,85
430.200632/1216.775	430,2006318	20,27957778	18,74
434.672277/1449.443	434,6722771	24,15738558	13,56
435.181292/1392.421	435,1812918	23,20701655	20,18
436.224365/1604.09	436,2243654	26,73483786	19,68

437.22758/1603.401	437,22758	26,72335754	17,54
438.677677/1763.369	438,6776767	29,38947692	15,76
442.195188/1213.07	442,1951882	20,21783167	17,66
444.678335/1389.577	444,6783355	23,15962007	14,81
445.234116/1131.271	445,234116	18,85450941	18,79
445.685726/1317.599	445,6857257	21,95997874	13,37
447.13451/1743.8	447,13451	29,06333393	18,09
448.120871/1977.551	448,1208713	32,95918744	17,53
451.199547/1640.605	451,1995466	27,3434211	16,94
451.698063/1344.384	451,6980627	22,40640803	15,51
453.752029/1209.719	453,7520288	20,1619817	14,68
456.630578/1929.795	456,6305782	32,16324359	19,55
457.133081/1929.812	457,1330812	32,16354081	17,98
457.635126/1929.771	457,6351265	32,16285129	16,27
461.71289/1220.865	461,7128898	20,34775286	13,90
462.190898/1220.712	462,1908982	20,34519762	17,51
462.706609/1339.049	462,7066092	22,31747984	14,64
464.684514/1212.616	464,684514	20,21026703	13,28
468.703106/1451.689	468,703106	24,19482171	14,32
470.225944/1653.39	470,225944	27,55650688	17,43
470.703741/1183.775	470,7037412	19,72958001	14,72
481.238133/1219.328	481,2381329	20,32213214	15,33
484.698435/1368.309	484,6984353	22,80514295	15,24
486.247369/1708.019	486,2473691	28,4669869	20,13
488.133087/1622.375	488,1330872	27,03958472	19,29
488.214118/1369.828	488,2141182	22,83047402	22,28
489.215511/1369.069	489,2155112	22,81781094	20,28
489.229227/1205.593	489,2292272	20,09322211	19,03
489.665135/1342.397	489,6651353	22,37328039	14,50
490.134078/1623.722	490,134078	27,06203208	16,47
490.21951/1368.282	490,21951	22,80470357	17,63
492.205293/1471.231	492,2052929	24,52051938	16,79
493.253588/1677.895	493,2535878	27,96491312	15,97
495.748052/1194.549	495,7480519	19,90914215	14,95
497.661528/1356.495	497,6615285	22,60824506	13,63
498.255692/1806.746	498,2556915	30,11242689	15,39
499.721026/1363.67	499,7210256	22,72782957	14,53
501.226919/1696.798	501,2269192	28,279971	16,28
502.224384/1707.009	502,2243842	28,45015414	16,28
506.200924/1752.808	506,2009238	29,2134718	16,15
515.24634/1400.671	515,2463404	23,34451668	15,51
515.248321/1729.411	515,2483211	28,82351853	16,06
518.231079/1746.072	518,2310791	29,10120139	15,29
518.249367/1644.683	518,2493668	27,41138073	14,93
518.730279/1191.19	518,7302786	19,8531684	14,93
519.202828/1716.55	519,2028283	28,60916124	17,18
519.261834/1679.677	519,261834	27,99461568	16,29
524.18742/1537.513	524,1874198	25,62521491	15,28
532.237327/1715.671	532,2373266	28,59451929	15,34
537.252966/1745.497	537,2529662	29,09161056	16,26
537.259601/1206.691	537,2596009	20,1115228	15,45
537.65526/1977.926	537,6552596	32,96542568	19,10

538.15717/1977.551	538,1571699	32,95918744	17,90
538.256933/1745.852	538,2569334	29,09753333	15,13
541.262762/1216.547	541,2627622	20,27579099	15,15
542.266486/1216.08	542,2664862	20,26799739	14,90
543.733933/1491.624	543,7339327	24,86039627	13,59
545.181617/1534.794	545,1816167	25,57989247	17,51
548.236652/1770.107	548,2366522	29,50178164	16,56
551.230866/1687.373	551,2308658	28,12287597	15,00
556.233638/1715.476	556,2336378	28,59126324	16,25
557.22316/1733.993	557,2231598	28,89988669	15,04
557.728983/1522.141	557,7289831	25,36900963	14,74
558.246165/1439.659	558,2461647	23,99431211	18,32
559.235568/1778.845	559,2355682	29,64742487	15,95
559.261777/1211.451	559,2617769	20,19084567	15,80
564.228867/1194.728	564,2288667	19,9121261	15,60
564.755231/1194.859	564,7552315	19,91431623	14,18
565.264247/1755.841	565,2642474	29,26401596	16,42
566.26682/1756.102	566,2668204	29,2683656	14,90
567.306418/1742.115	567,3064183	29,03524965	18,11
568.275617/1738.142	568,2756168	28,9690395	17,85
570.254059/1734.858	570,2540592	28,91429373	15,80
570.736694/1175.967	570,7366943	19,5994569	15,63
571.12954/1927.947	571,1295405	32,13245331	13,78
571.233572/1187.381	571,2335721	19,78968591	17,25
571.75515/1187.613	571,7551504	19,79354428	15,92
572.247057/1190.971	572,2470572	19,84951362	16,04
574.245882/1803.627	574,245882	30,06045483	15,62
575.230134/1758.306	575,2301345	29,30509964	14,74
581.170349/2003.087	581,1703491	33,38478874	15,85
581.261495/1791.803	581,2614954	29,86337724	16,17
582.245846/1711.533	582,2458461	28,52555629	15,55
582.261043/1789.684	582,2610432	29,82807281	15,32
583.284852/1764.808	583,2848516	29,41346918	15,38
584.253179/1203.079	584,2531791	20,05132495	15,30
593.28699/1271.06	593,28699	21,18432928	13,91
594.258391/1285.899	594,2583907	21,43164897	16,89
594.67484/2007.316	594,6748398	33,45526924	14,17
595.274204/1800.156	595,2742037	30,00259454	15,46
597.298388/1782.142	597,2983879	29,70237409	14,01
598.243112/1817.154	598,2431123	30,28589325	14,82
599.157187/2007.515	599,1571868	33,45858828	18,10
599.254308/1775.366	599,254308	29,58942879	14,82
599.294007/1215.768	599,294007	20,26279527	14,74
599.745744/1190.383	599,745744	19,83972129	15,56
599.761164/2005.377	599,7611635	33,42294345	15,66
600.686619/2008.125	600,6866186	33,46875825	13,92
600.737245/1190.894	600,7372445	19,84823332	13,56
602.240525/1285.603	602,2405248	21,42672213	16,22
603.25773/1872.357	603,2577304	31,20595449	16,31
607.287674/1266.867	607,2876741	21,11445337	17,24
613.281398/1205.717	613,2813976	20,09527859	14,73
614.292271/1810.528	614,2922713	30,17547232	15,77

614.2963/1730.712	614,2963001	28,84519658	14,01
620.247793/1820.414	620,2477933	30,34023657	14,89
627.195496/1743.897	627,1954956	29,0649441	21,08
628.198697/1743.893	628,1986965	29,06487812	19,08
629.20105/1743.897	629,2010498	29,0649441	17,17
630.204347/1743.893	630,2043469	29,06487812	14,68
630.329301/1777.574	630,3293012	29,62623844	14,54
633.245859/1808.377	633,2458589	30,1396222	14,01
638.284268/1843.128	638,2842685	30,71880022	13,96
639.191648/2019.722	639,1916478	33,66203958	14,90
645.302376/1782.53	645,3023764	29,70884146	13,61
647.769309/1214.697	647,7693085	20,24494297	12,80
649.261459/1503.246	649,2614586	25,0540934	15,08
650.18319/1749.625	650,1831897	29,1604185	14,96

Supplementary Table S3: Clinical characteristics of UPJ discovery cohort

Sample code	Clinical characteristics			Gender	Age (days)	Cohort
	Status	HN grade	Pelvis dilatation			
C63-1-14	Healthy control			M	1	discovery
C63-1-7	Healthy control			M	1	discovery
C63-1-3	Healthy control			M	2	discovery
C63-1-8	Healthy control			M	2	discovery
C63-1-4	Healthy control			M	3	discovery
C63-1-13	Healthy control			M	4	discovery
C63-1-5	Healthy control			M	5	discovery
C30-4-3	Healthy control			M	48	discovery
C47-1-2	Healthy control			M	94	discovery
C30-3-16	Healthy control			M	104	discovery
C59-1-5	Healthy control			M	123	discovery
C63-1-16	Healthy control			M	143	discovery
C63-2-7	Healthy control			M	152	discovery
C63-2-5	Healthy control			M	176	discovery
C47-1-3	Healthy control			M	187	discovery
C22-2-2	UPJ obstruction	3	16	M	38	discovery
C11-4-14	UPJ obstruction	3	21	M	44	discovery
C42-4-11	UPJ obstruction	3	25	M	44	discovery
C42-4-4	UPJ obstruction	3	26	F	37	discovery
C49-4-12	UPJ obstruction	3	43	M	34	discovery
C42-4-14	UPJ obstruction	4	17	M	29	discovery
C3-1-1	UPJ obstruction	4	22	M	53	discovery
C7-1-20	UPJ obstruction	4	22	M	53	discovery
C5-3-11	UPJ obstruction	4	23	M	33	discovery
C2-3-13	UPJ obstruction	4	24	M	37	discovery
C49-3-4	UPJ obstruction	4	24	M	106	discovery
C63-2-2	UPJ obstruction	4	25	M	50	discovery
C16-4-15	UPJ obstruction	4	25	M	58	discovery
C58-2-6	UPJ obstruction	4	26	M	30	discovery
C57-1-21	UPJ obstruction	4	28	F	56	discovery
C63-2-3	UPJ obstruction	4	30	M	20	discovery
C11-3-17	UPJ obstruction	4	30	M	26	discovery
C58-4-9	UPJ obstruction	4	30	M	39	discovery
C55-1-5	UPJ obstruction	4	31	M	40	discovery
C31-1-17	UPJ obstruction	4	33	M	39	discovery
C17-3-14	UPJ obstruction	4	34	M	58	discovery
C16-3-3	UPJ obstruction	4	35	M	40	discovery
C50-1-6	UPJ obstruction	4	50	M	20	discovery
C50-2-1	UPJ obstruction	4	50	M	36	discovery
C5-3-6	UPJ obstruction	ND	25	M	35	discovery
C6-3-16	UPJ obstruction	ND	16	M	167	discovery
C63-2-4	UPJ obstruction	ND	20	M	133	discovery
C6-3-19	UPJ obstruction	ND	22	M	112	discovery
C5-3-10	UPJ obstruction	ND	30	M	213	discovery

C6-3-17	UPJ obstruction	ND	34	M	105	discovery
C6-4-11	UPJ obstruction	ND	40	M	69	discovery
C5-3-1	UPJ obstruction	ND	ND	F	80	discovery
C5-4-15	UPJ obstruction	ND	ND	M	174	discovery
C6-4-21	UPJ obstruction	ND	ND	M	193	discovery

HN: Hydronephrosis. ND: undetermined.

Supplementary Table S4: Clinical characteristics of UPJ validation cohort

Sample code	Score UPJMetab32	Clinical characteristics			Gender	Age (days)	Cohort
		Status	HN grade	Pelvis dilatation			
C30-4-5	-0,1674	Healthy control			M	18	validation
C63-1-10	-1,0277	Healthy control			M	1	validation
C63-1-2	-0,9342	Healthy control			M	1	validation
C63-1-1	-0,4827	Healthy control			M	1	validation
C63-1-9	-0,7862	Healthy control			M	4	validation
C30-4-2	0,4222	Healthy control			M	261	validation
C63-1-6	-0,2766	Healthy control			M	1	validation
C2-3-1	-0,3163	UPJ obstruction	ND	15	M	193	validation
C5-4-5	0,0726	UPJ obstruction	ND	15	M	232	validation
C5-3-17	0,9125	UPJ obstruction	ND	16	M	153	validation
C28-4-12	1,0583	UPJ obstruction	3	17	M	49	validation
C47-3-10	0,9652	UPJ obstruction	4	19	M	47	validation
C47-1-10	0,5623	UPJ obstruction	4	20	M	64	validation
C5-3-20	-0,0424	UPJ obstruction	4	23	M	196	validation
C55-2-11	0,4952	UPJ obstruction	4	23	F	42	validation
C44-4-18	0,2127	UPJ obstruction	4	25	M	69	validation
C9-2-7	-0,0509	UPJ obstruction	3	27	F	27	validation
C7-1-18	1,1222	UPJ obstruction	4	31	M	29	validation
C31-4-10	0,8644	UPJ obstruction	4	33	M	33	validation
C3-1-15	0,3647	UPJ obstruction	4	37	M	24	validation
C50-1-20	1,0617	UPJ obstruction	4	46	F	29	validation
C55-1-1	0,6135	UPJ obstruction	4	50	M	36	validation
C56-1-2	0,5371	UPJ obstruction	4	67	M	60	validation
C5-3-19	-0,510	UPJ obstruction	ND	ND	M	262	validation

Supplementary Table S5: List of 32 urinary metabolites differentially excreted between healthy and UPJ patients in the discovery cohort.

ID	Raw-intensity			Log2-intensity		p-value	
	Healthy	UPJ obstruction	FC	Healthy	UPJ obstruction	Wilcoxon	BH
324.083234/1512.623	708506	406112	0,57	19,34	18,56	1,8E-05	0,0035
456.630578/1929.795	3173450	1588974	0,50	21,50	20,26	1,8E-05	0,0035
177.031281/1357.129	526987	318408	0,60	18,92	18,20	1,3E-04	0,0135
366.599792/1929.853	788580	605801	0,77	19,54	18,89	1,4E-04	0,0135
227.111791/989.758	10196251	24720725	2,42	22,67	24,26	2,5E-04	0,0164
467.617961/1929.795	129782	73106	0,56	16,83	15,99	2,5E-04	0,0164
457.133081/1929.812	864131	540265	0,63	19,58	18,91	3,0E-04	0,0166
438.677677/1763.369	83802	53536	0,64	16,26	15,63	6,7E-04	0,0182
490.134078/1623.722	114760	85880	0,75	16,76	16,30	8,0E-04	0,0182
157.077555/1067.986	198756	369487	1,86	17,56	18,25	7,9E-04	0,0193
242.074921/1533.731	3719793	7549206	2,03	21,63	22,61	5,9E-04	0,0193
299.056259/1357.852	500388	304343	0,61	18,82	18,10	7,4E-04	0,0193
338.098419/1482.724	633012	468298	0,74	19,20	18,81	5,8E-04	0,0193
390.245275/1571.028	905682	401355	0,44	19,42	18,07	3,3E-03	0,0193
474.701959/1359.882	132854	80873	0,61	16,85	16,22	1,4E-03	0,0193
488.133087/1622.375	1150184	811380	0,71	20,07	19,55	5,8E-04	0,0193
229.117309/1322.695	108917815	204227951	1,88	26,49	27,30	9,3E-04	0,0194
234.129064/1411.802	230142	158724	0,69	17,72	17,20	1,7E-03	0,0194
368.181291/1241.422	219228	127700	0,58	17,58	16,85	2,4E-03	0,0194
632.28172/1223.924	166033	115919	0,70	17,26	16,49	3,7E-03	0,0194
211.087719/991.444	88269	164219	1,86	16,27	17,10	1,4E-03	0,0240
228.167817/1066.426	64195	110790	1,73	15,75	16,46	1,4E-03	0,0240
237.193077/1281.495	96751	187287	1,94	16,28	17,26	1,5E-03	0,0240
526.161131/1357.627	432409	272417	0,63	18,56	17,72	1,2E-03	0,0240
181.105924/990.207	732059	1324472	1,81	19,03	20,14	2,3E-03	0,0333
228.114334/990.108	894846	1902390	2,13	19,20	20,55	2,1E-03	0,0344
259.090975/1390.694	1117825	485984	0,43	19,48	18,46	3,8E-03	0,0356
306.619622/1015.236	45530	67407	1,48	15,35	15,92	6,0E-03	0,0366
303.615001/1045.311	16068	24344	1,52	13,91	14,41	1,4E-03	0,0407
355.071351/1117.064	547815	283969	0,52	18,80	17,97	4,0E-03	0,0424
537.65526/1977.926	4714555	2813304	0,60	21,96	20,87	1,2E-02	0,0435
185.125274/1291.16	165754	317070	1,91	17,18	17,79	2,3E-03	0,0481

FC: fold change between UPJ obstruction and healthy patients. BH: Benjamini and Hochberg.
